# Socioeconomic disadvantage in childhood as a predictor of excessive gestational weight gain and obesity in midlife adulthood

**DOI:** 10.1186/s12982-015-0026-7

**Published:** 2015-03-06

**Authors:** Benjamin W Chaffee, Barbara Abrams, Alison K Cohen, David H Rehkopf

**Affiliations:** Division of Oral Epidemiology and Dental Public Health, University of California San Francisco, San Francisco, USA; Division of Epidemiology and Division of Community Health and Human Development, School of Public Health, University of California Berkeley, Berkeley, USA; Division of Epidemiology, School of Public Health, University of California Berkeley, Berkeley, USA; Division of General Medical Disciplines, Stanford University, Stanford, USA

**Keywords:** Gestational weight gain, Obesity, Social class, Socioeconomic factors

## Abstract

**Background:**

Lower childhood socioeconomic position is associated with greater risk of adult obesity among women, but not men. Pregnancy-related weight changes may contribute to this gender difference. The objectives of this study were to determine the associations between: 1. childhood socioeconomic disadvantage and midlife obesity; 2. excessive gestational weight gain (GWG) and midlife obesity; and 3. childhood socioeconomic disadvantage and excessive GWG, among a representative sample of childbearing women.

**Methods:**

We constructed marginal structural models for seven measures of childhood socioeconomic position for 4780 parous women in the United States, using National Longitudinal Survey of Youth (1979–2010) data. Institute of Medicine definitions were used for excessive GWG; body mass index ≥30 at age 40 defined midlife obesity. Analyses were separated by race/ethnicity. Additionally, we estimated controlled direct effects of childhood socioeconomic disadvantage on midlife obesity under a condition of never gaining excessively in pregnancy.

**Results:**

Low parental education, but not other measures of childhood disadvantage, was associated with greater midlife obesity among non-black non-Hispanic women. Among black and Hispanic mothers, childhood socioeconomic disadvantage was not consistently associated with midlife obesity. Excessive GWG was associated with greater midlife obesity in all racial/ethnic groups. Childhood socioeconomic disadvantage was not statistically significantly associated with excessive GWG in any group. Controlled direct effects were not consistently weaker than total effects.

**Conclusions:**

Childhood socioeconomic disadvantage was associated with adult obesity, but not with excessive gestational weight gain, and only for certain disadvantage measures among non-black non-Hispanic mothers. Prevention of excessive GWG may benefit all groups through reducing obesity, but excessive GWG does not appear to serve as a mediator between childhood socioeconomic position and adult obesity in women.

## Background

Compelling evidence suggests that low childhood socioeconomic position (SEP) is associated with high levels of adult obesity among women, even after controlling for adult SEP [1]. However, few studies reporting this finding have included diverse populations, limiting the ability to examine differences by race/ethnicity [1]. Furthermore, an observed gender nonconformity in obesity differences by SEP raises questions as to what weight-related risk factors might mediate the relationship between childhood SEP and adult obesity among women but not men [1]. Uncovering such pathways is critical to guide actions to reduce any excess obesity among women attributable to low childhood SEP. Excessive gestational weight gain (GWG) is a highly prevalent condition [2] that contributes to obesity later in life [3]. Thus, excessive GWG represents a candidate mediator between early-life disadvantage and obesity in childbearing females. Regardless of any potential mediating role, excessive GWG itself has important ramifications for the health of women and children [2,4,5], and better understanding of contributing factors could inform prevention.

No previous studies have considered the association between childhood SEP and gestational weight gain or the role of excessive GWG as a possible mediator between childhood SEP and adult obesity. We first aimed to estimate the association between early-life socioeconomic disadvantage and obesity at midlife among parous women in the National Longitudinal Survey of Youth (NLSY) 1979 cohort [6], using multiple of markers of childhood SEP, including parental educational attainment, parental employment, and household income. We next aimed to estimate the associations between excessive GWG and midlife obesity and, lastly, between low early-life SEP and excessive GWG. Secondarily, to examine mediation by excessive GWG between childhood socioeconomic disadvantage and adult obesity, we estimated the childhood SEP-obesity association under a condition in which no woman gains excessively in pregnancy (i.e. controlled direct effect).

## Methods

**Figure 1:**
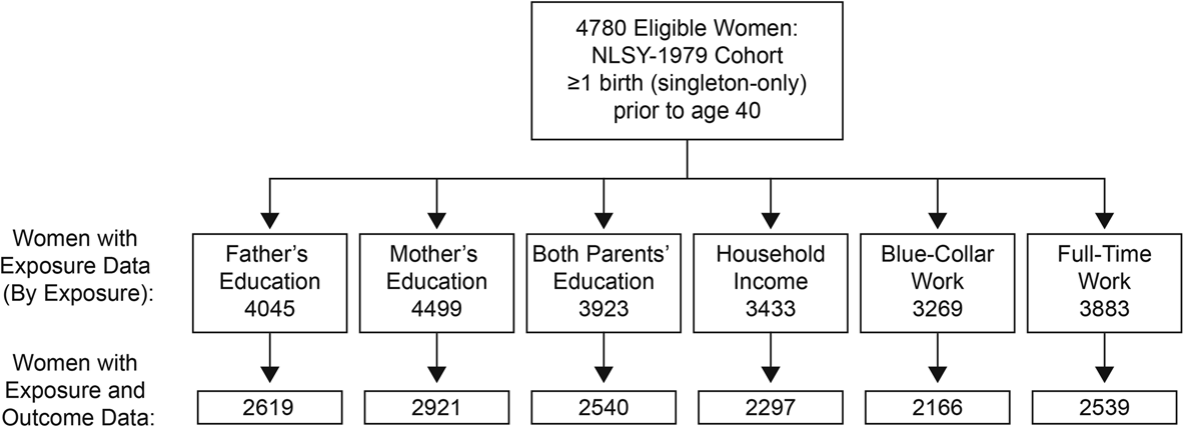
**Eligible women included in the analysis by measure of childhood SEP (exposure) and midlife obesity (outcome).** Legend: The figure shows the number of eligible women included in the analysis in separate models for each of seven different measures of childhood socioeconomic position. Exposure to household income below 100% and 200% of the federal poverty level was available for the same sample. Missing gestational weight gain data (GWG) were imputed.

Using a complex multistage sampling design, the NLSY 1979 cohort recruited men and women, age 14–21 years in 1979, and has followed them since through in-person and telephone interviews [6]. Pregnancy data were collected beginning in 1986: pregnancies prior to 1986 were recorded in that survey year, with subsequent pregnancies recorded prospectively [6]. We excluded women with non-singleton births, and, to maintain temporal ordering, considered only births before age 40, the age at which the main outcome (obesity) was recorded. This yielded 4,780 eligible women, who had 10,908 births (Figure 1).

**Figure 2:**
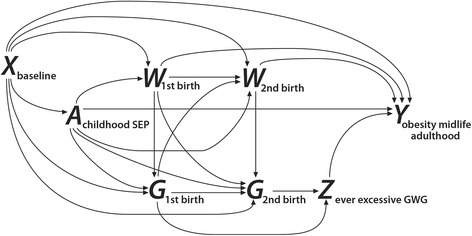
**Relationships among study variables.** Legend: The diagram depicts the assumed relationships between the types of variables considered in this analysis of data from the National Longitudinal Survey of Youth 1979 Cohort, United States, 1979–2010. The diagram depicts women with two births as an example to show time-dependencies between variables over multiple births. X = early-life family situation (e.g. immigrant status, urban residence, region of country); A = childhood socioeconomic position (i.e. exposure); W = birth-specific (time-varying) maternal variables (e.g. marital status, household income, age at birth, pre-pregnancy body mass index); G = gestational weight gain for each birth; Z = lifetime history (ever vs. never) of excessive gestational weight gain (i.e. mediator); Y = midlife obesity (i.e. outcome). All directed paths including A-Z-Y represent the indirect effect of A on Y mediated by Z. The direct effect of A on Y includes all directed paths from A to Y that do not include Z.

Following notation of VanderWeele [7], we considered six types of variables (Figure 2), described below. Outcome (Y) was obesity at midlife, defined as body mass index (BMI) ≥30 kg/m^2^ at age 40 or 41 (collected 2002–2010). Exposure (A) was early-life socioeconomic disadvantage, as reflected by baseline SEP. We used seven different binary variables to categorize disadvantage in separate models: education of the respondent’s father <12 years; education of the respondent’s mother <12 years; education of both parents <12 years; income of household where the respondent lived as a dependent in 1978 < 200% of federal poverty level; or <100% of federal poverty level; respondent’s father/stepfather worked in a blue-collar occupation in 1979; and respondent’s father/stepfather worked less than full-time in 1979 (part-time or unemployed). Variables were chosen to provide multiple measures of early life socioeconomic position, given that the relationship between SEP and health outcomes often differs depending on the factor considered [8]. We dichotomized exposure variables in part due to what would be a limited number of observations in some SEP categories when stratified by race/ethnicity (e.g. few parents of black and Hispanic NLSY respondents attained ≥16 years of education). The putative mediator (Z) was history of ever experiencing ≥1 excessive GWG event in any birth prior to age 40 versus never having gained excessively. Total GWG was calculated for each birth by subtracting self-reported pre-pregnancy weight from self-reported delivery weight and then categorizing the GWG according to the 2009 Institute of Medicine guidelines [2]. Excessive GWG was defined as weight gain above the guideline upper limit, based on pre-pregnancy body mass index (BMI).

We restricted estimates of the A ~ Y and A ~ Z associations to women with measured exposure information (Figure 1), rather than imputing missing childhood experiences. GWG data were available for 9,347 of 10,908 births (85.7%), and ever-never GWG status was known for 4,124 of 4,780 women (86.3%). Compared to exposure variables, GWG data had fewer missing values and a richer set of predictors; thus, we multiply imputed missing GWG data at the level of each pregnancy in order to assign missing ever-never excessive GWG status. As a sensitivity check, we repeated all analyses restricted only to those women with measured GWG data for all recorded births, which did not appreciably alter estimates.

We differentiated between confounders of the A ~ Y and A ~ Z relationships (denoted X) and confounders of the Z ~ Y relationship (denoted W), both determined a priori (Figure 2). X confounders were all binary individual-specific variables: birth outside the US, urban residence as a child, and residence in the South as a child; but excluded variables plausibly on a causal path between childhood SEP and adult obesity (e.g. adult SEP, pre-pregnancy BMI). W confounders included all X confounders, plus additional birth-specific and potentially time-varying maternal variables: age, marital status, smoking during pregnancy, educational attainment (<12, 12–15, ≥16 years), pre-pregnancy BMI (linear and quadratic terms), equivalized household income [9], and previous excessive or inadequate GWG. Missing values for X and W confounders were addressed using multiple imputation. Race/ethnicity (denoted V) was defined as non-Hispanic black, non-black Hispanic, and non-black non-Hispanic (of which 98.4% self-identified as white). We present separate results by race/ethnicity, because early-life socioeconomic factors, as measured by educational attainment, for example, may have different associations with adult health by race/ethnicity [10,11].

Using the potential outcomes framework, we refer to Y_a1_ as an individual’s midlife obesity status had, possibly counter-to-fact, her early-life SEP been disadvantaged, and Y_a0_ as that same individual’s midlife obesity status had, possibly counter-to-fact, her early-life SEP been not disadvantaged. Averaged over the population, we define the cumulative incidence ratio (risk ratio, RR) for midlife obesity under these two settings of early-life SEP as: E[Y_a1_/Y_a0_]. We had seven measures of early-life SEP and three race/ethnicity subgroups, and, therefore, were interested in 21 different A ~ Y associations. Additionally, we wished to estimate the effect of ever experiencing excessive GWG on midlife obesity: E[Y_z1_/Y_z0_], in each race/ethnicity subgroup, as well as the effect of early-life socioeconomic disadvantage on ever experiencing excessive GWG: E[Z_a1_/Z_a0_], for each of our seven SEP measures and three race/ethnicity subgroups.

We estimated the above parameters using marginal structural models (MSM) and inverse probability of treatment weighting estimators [12-14]. Briefly, observations were up-weighted that, based on covariates, were less likely to obtain their observed exposure status. The weighting provides balance across the exposed and unexposed populations with respect to the confounding variables used to estimate the weights. In this weighted “pseudo-population,” the adjusted exposure-outcome association carries a population-average interpretation. Causal inference requires further assumptions: consistency, positivity, exchangeability, and correct specification of the treatment models that generated the weights [14,15]. We also assume correct specification of the imputation model specification and missingness at random. We specifically refer to our results as “associations” rather than “effects” to emphasize the strength of these assumptions.

Weights were the inverse probability of observed exposure status, given putative confounding variables. We obtained these probabilities from regression models for the exposure, using general estimating equations with exchangeable correlation structure (clustering on household). For estimating the Z ~ Y association, where the “exposure” was ever experiencing excessive GWG, exposure probability was based on estimating the probability of excessive GWG in observed births (clustered within women) and then using chain probabilities for ever-never status, which allowed for time-varying W confounders, such as age and previous excessive GWG events. Using notation from VanderWeele [16], for each individual *i*, the exposure weights for A $\left({w}_i^A\right)$ and for Z $\left({w}_i^Z\right)$ were

$$ {w}_i^A=\frac{P\left(A={a}_i\Big|V={v}_i\right)}{P\left(A={a}_i\Big|X={x}_i,V={v}_i\right)} $$

and

$$ {w}_i^Z=\frac{P\left(Z={z}_i\mathit{\Big|}V={v}_i\right)}{P\left(Z={z}_i\mathit{\Big|}A={a}_i,X={x}_i,W={w}_i,V={v}_i\right)} $$

Having seven measures of early-life SEP, we estimated seven MSMs for the A ~ Y association, seven MSMs for the A ~ Z association, as well as one MSM for the Z ~ Y association. All MSMs were log-linear models and included interaction terms for race-ethnicity (V).

To assess potential mediation by excessive GWG, we estimated controlled direct effects following methodology proposed by VanderWeele [7]. Pearl [17] defines the controlled direct effect as the effect of exposure on outcome under a hypothetical intervention to hold the mediator at a specific value. Of interest was the associations between early-life SEP and midlife obesity if excessive GWG were prevented in all pregnancies, or E[Y_a1z0_ – Y_a0z0_]. If this controlled direct effect is reduced in magnitude with respect to the total effect, this suggests that the potential mediator is part of a pathway between exposure and outcome [7]. The controlled direct effect is estimated from an MSM containing exposure weights for both A and Z, and model terms for A, Z, and A-by-Z interaction [7].

All inverse probability weights were stabilized [14]. In order to reduce the variability of estimates, final weights were truncated at the 1st and 99th percentiles [14]. In addition to exposure and mediator weights, as appropriate, all MSMs included NLSY baseline (year 1979) sampling weights to be nationally representative. We included inverse probability censoring weights to account for losses to follow-up [14]. Variables in the censoring models were childhood SEP measures and all X and W confounders. Point estimates were averaged over 25 multiple imputations. We used bootstrap re-sampling of households to estimate 95% confidence intervals (at the 0.025 and 0.975 quantile). Associations were considered statistically significant if 95% confidence intervals for the risk ratio excluded one. We did not adjust for multiple comparisons [18]. Analyses were completed in R version 3.1.0 (http://www.r-project.org/). The University of California Berkeley Committee for Protection of Human Subjects did not consider this study human subjects research because data were de-identified and openly available on the Internet.

## Results

The prevalence of midlife obesity was highest among non-Hispanic black mothers (49.2%), compared to non-black Hispanic mothers (38.6%) and non-black non-Hispanic mothers (26.4%) (Table 1). Childhood socioeconomic disadvantage varied in prevalence depending on the measure of disadvantage considered but was more common among racial/ethnic minorities than among non-black non-Hispanic mothers (Table 1). Excessive gestational weight gain was observed in 43.8% of pregnancies, and 62.8% of mothers experienced excessive GWG at least once.

**Table 1:** **Characteristic of Eligible Parous Women in National Longitudinal Survey of Youth 1979 Cohort, 1979-2010**

	**Overall**	**Non-Black, Non-Hispanic**	**Black, Non-Hispanic**	**Non-Black, Hispanic**
Women (n at baseline)^a,b^	4780	2902	1204	674
Childhood socio-economic position				
Father education <12 yrs, %	37.9	32.6	53.7	72.8
Mother education <12 yrs, %	36.3	29.8	54.6	81.1
Both Father and Mother education <12 yrs, %	23.3	17.4	39.2	66.9
Dependent in household earning <200% of federal poverty level, %	38.4	29.4	75.8	70.5
Dependent in household earning <100% of federal poverty level, %	15.4	8.3	45.5	39.3
Father/stepfather had blue-collar occupation, %	61.3	56.1	80.9	85.5
Father/stepfather employed less than full-time, %	17.2	14.9	25.4	28.7
Maternal characteristics				
Foreign born, %	4.6	3.5	2.1	26.6
Urban residence as child, %	78.1	76.8	81.4	87.7
<12 yrs formal education, %	18.4	15.3	27.0	39.3
<16 yrs formal education, %	80.6	77.5	90.4	91.1
Number of reported births (SD)	2.3 (1.0)	2.2 (1.1)	2.5 (1.3)	2.7 (1.3)
Age at first birth, yrs (SD)	23.8 (5.5)	24.5 (5.4)	21.1 (5.1)	22.0 (5.0)
Maternal pregnancy-related weight				
BMI prior to first pregnancy (SD)	22.4 (4.3)	22.4 (4.2)	22.6 (4.3)	22.6 (3.8)
Ever gained excessively in pregnancy^c^, %	62.8	62.4	61.4	72.2
Ever overweight (BMI ≥25) prior to pregnancy, %	26.1	24.3	31.8	37.4
Ever obese (BMI ≥30) prior to pregnancy, %	13.6	12.5	18.4	16.9
Obese at age 40, %	30.4	26.4	49.2	38.6

^a^Baseline sampling weights used to obtain nationally representative estimates.

^b^Sample sizes may differ by characteristic due to missing data.

^c^For women with incomplete ever-never excessive gestational weight gain history (13.7%), missing data were imputed for individual births and averaged over 25 imputations.

Abbreviations: BMI = body mass index; SD = standard deviation.

**Figure 3:**
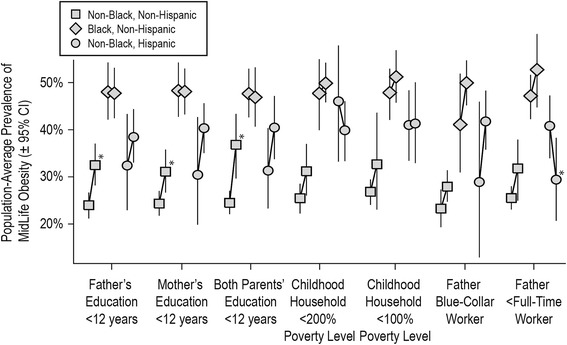
**Estimated Population-Average Effect of Childhood Socioeconomic Adversity on Midlife Obesity.** Legend: Each pair of symbols represents the adjusted prevalence of obesity (BMI ≥30) at age 40–41 among parous women in the NLSY 1979–2010 cohort under two conditions: had the entire cohort experienced childhood socioeconomic adversity (right symbol), or had no one in the cohort experienced childhood socioeconomic adversity (left symbol), from seven different marginal structural models based on seven different indicators of socioeconomic position. * = *P* < 0.05; CI = confidence interval.

All seven measures of childhood socioeconomic disadvantage were associated with a higher prevalence of midlife obesity among non-black non-Hispanic mothers (Figure 3, Table 2), but only for parental education variables were these associations statistically significant. There were no statistically significant associations among non-Hispanic black mothers, although obesity prevalence was greater with paternal blue-collar occupation and with paternal less than full-time employment. Among non-black Hispanic mothers, low parental education and paternal blue-collar occupation were associated with a higher prevalence of midlife obesity, albeit not statistically significant, while household income <200% of the federal poverty level and paternal less than full-time employment were associated with lower obesity prevalence, the latter association being statistically significant.

**Table 2:** **Estimated Population-Average Effect of Childhood Socioeconomic Adversity on Midlife Obesity and Excessive Gestational Weight Gain**

	**Outcome: Midlife Obesity**			**Outcome: Excessive Gestational Weight Gain**		
**Childhood socio-economic position**	**Non-Black, Non-Hispanic**	**Black, Non-Hispanic**	**Non-Black, Hispanic**	**Non-Black, Non-Hispanic**	**Black, Non-Hispanic**	**Non-Black, Hispanic**
	**RR (95% CI)**	**RR (95% CI)**	**RR (95% CI)**	**RR (95% CI)**	**RR (95% CI)**	**RR (95% CI)**
Father education <12 years	1.4*	1.0	1.2	1.0	1.0	1.1
	(1.1, 1.6)	(0.8, 1.2)	(0.9, 1.7)	(1.0, 1.1)	(0.9, 1.1)	(0.9, 1.2)
Mother education <12 years	1.3*	1.0	1.3	1.0	1.0	1.1
	(1.1, 1.6)	(0.9, 1.2)	(0.9, 2.1)	(1.0, 1.1)	(0.9, 1.1)	(0.9, 1.3)
Both Father and Mother education <12 years	1.5*	1.0	1.3	1.0	1.0	1.1
	(1.2, 1.8)	(0.8, 1.2)	(0.9, 1.8)	(1.0, 1.1)	(0.9, 1.2)	(1.0, 1.3)
Dependent in household earning <200% of federal poverty level	1.2	1.0	0.9	1.0	0.9	1.0
	(1.0, 1.6)	(0.9, 1.3)	(0.7, 1.2)	(0.9, 1.1)	(0.8, 1.0)	(0.9, 1.2)
Dependent in household earning <100% of federal poverty level	1.2	1.1	1.0	1.0	1.0	0.9
	(0.9, 1.7)	(0.9, 1.3)	(0.8, 1.4)	(0.9, 1.1)	(0.9, 1.3)	(0.8, 1.1)
Father/stepfather had blue-collar occupation	1.2	1.2	1.4	1.0	1.1	0.9
	(1.0, 1.5)	(0.9, 1.6)	(0.9, 2.8)	(0.9, 1.1)	(0.9, 1.2)	(0.8, 1.0)
Father/stepfather employed less than full-time	1.2	1.1	0.7*	1.1	0.9	1.0
	(1.0, 1.5)	(0.9, 1.3)	(0.5, 1.0)	(0.9, 1.2)	(0.8, 1.0)	(0.9, 1.2)

Table shows the risk ratios for parous women in the NLSY 1979–2010 cohort for two outcomes: obesity (BMI ≥30) at age 40–41 (left columns) and lifetime history of ever experiencing excessive gestational weight gain (right columns), comparing two exposure conditions: had the entire cohort experienced childhood socioeconomic adversity or had no one in the cohort experienced childhood socioeconomic adversity, from seven different marginal structural models for seven different indicators of socioeconomic position.

* = *P* < 0.05.

Abbreviations: CI = confidence interval; RR = risk ratio (cumulative incidence ratio).

Ever experiencing excessive GWG was associated with a higher prevalence of midlife obesity in all three racial/ethnic groups: non-black, non-Hispanic (RR: 1.5; 95% CI: 1.2, 2.0), non-Hispanic black (RR: 1.4; 95% CI: 1.2, 1.8), and non-black Hispanic (RR: 1.3; 95% CI: 0.9, 2.1), although not statistically significant among non-black Hispanic mothers.

**Figure 4:**
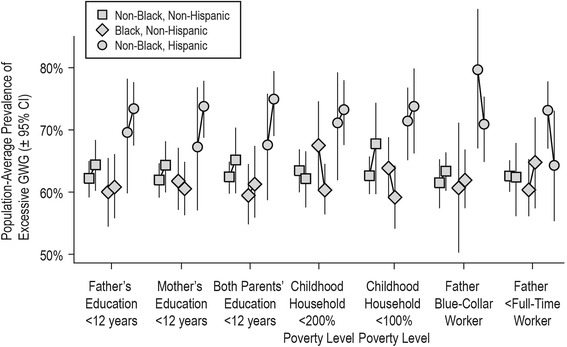
**Estimated Population-Average Effect of Childhood Socioeconomic Adversity on Excessive Gestational Weight Gain.** Legend: Each pair of symbols represents the adjusted prevalence of lifetime history of ever experiencing excessive gestational weight gain among parous women in the NLSY 1979–2010 cohort under two conditions: had the entire cohort experienced childhood socioeconomic adversity (right symbol), or had no one in the cohort experienced childhood socioeconomic adversity (left symbol), from seven different marginal structural models based on seven different indicators of socioeconomic position. CI = confidence interval; GWG = gestational weight gain.

None of the measures of childhood socioeconomic disadvantage were associated with ever experiencing excessive GWG to a statistically significant extent in any racial/ethnic group (Figure 4, Table 2). Of these non-statistically significant associations, the strongest positive associations occurred with low parental educational attainment among non-black Hispanic mothers. Other measures, such as low household income among non-Hispanic black mothers, and less than full-time paternal employment and blue-collar paternal occupation among non-black Hispanic mothers had inverse associations with excessive GWG.

There was no consistent evidence of mediation between childhood SEP and midlife obesity on the part of excessive GWG (Table 3). Had excessive GWG been a strong mediator, any association between childhood SEP and midlife obesity would be expected to be weakened under the condition of setting excessive GWG status to zero for all women. In contrast, the statistically significant and positive total associations of previous generation’s education on midlife obesity seen among non-black non-Hispanic women (Table 2) remained positive and statistically significant when estimated as controlled direct effects under a setting of never experiencing excessive GWG (Table 3).

**Table 3:** **Estimated Population-Average Risk for Midlife Obesity by Childhood Socioeconomic Position, Conditional on Never Experiencing Excessive Gestational Weight Gain**

**Childhood socio-economic position**	**Non-Black, Non-Hispanic**	**Black, Non-Hispanic**	**Non-Black, Hispanic**
	**RR (95% CI)**	**RR (95% CI)**	**RR (95% CI)**
Father education <12 years	1.7*	1.2	1.3
	(1.2, 2.5)	(0.8, 2.0)	(0.6, 3.7)
Mother education <12 years	1.5*	1.1	0.9
	(1.0, 2.2)	(0.7, 1.6)	(0.5, 3.0)
Both Father and Mother education <12 years	1.9*	1.1	1.1
	(1.3, 2.8)	(0.7, 1.7)	(0.5, 2.8)
Dependent in household earning <200% of federal poverty level	1.1	1.1	0.9
	(0.7, 1.9)	(0.8, 1.9)	(0.3, 2.5)
Dependent in household earning <100% of federal poverty level	0.9	1.1	1.2
	(0.4, 1.7)	(0.8, 1.8)	(0.4, 3.1)
Father/stepfather had blue-collar occupation	1.3	1.2	1.3
	(0.8, 2.0)	(0.6, 3.3)	(0.4, 4.5)
Father/stepfather employed less than full-time	1.2	1.3	0.5
	(0.6, 1.9)	(0.8, 1.9)	(0.2, 1.4)

Table shows the adjusted relative risks and risk differences for midlife obesity (BMI ≥30) at age 40–41 among parous women in the NLSY 1979–2010 cohort comparing two exposure states: had the entire cohort experienced childhood socioeconomic adversity or had no one in the cohort experienced childhood socioeconomic adversity, under the condition of never experiencing excessive gestational weight gain applied to the entire population. Results draw from seven different marginal structural models for seven different indicators of socioeconomic position.

* = *P* < 0.05.

Abbreviation: CI = confidence interval.

## Discussion

Our results suggest that different aspects of childhood SEP carry different implications for obesity in midlife, which may vary by race/ethnicity. Importantly, we confirmed that experiencing excessive GWG is associated with midlife obesity in multiple racial/ethnic groups. We found no measure of childhood socioeconomic disadvantage that was statistically significantly associated with excessive GWG. Our findings imply that it is highly unlikely that excessive GWG mediates a pathway between childhood SEP and adult obesity, and thus does not represent an opportunity to mitigate the effects of childhood SEP on adult obesity.

Race/ethnicity was an effect measure modifier for the childhood SEP - adult obesity relationship. We conceptualized race/ethnicity as a social, not a biological, construct, and from this can speculate about reasons for differences in SEP-obesity associations. There may be several ways in which the social indicators examined differ in their meaning by racial/ethnic group. Historical differences in school quality for blacks and whites [11], as well as differences in income returns to education, could explain how race/ethnicity modifies the effect of educational attainment on health [19]. This may account for why we saw no association between parental educational attainment and adult weight status for black women: for their parents’ generation, educational attainment was somewhat decoupled from life chances that could affect their children’s upbringing.

Congruent with previous studies [20,21], our findings suggest that associations between childhood SEP and adult weight status can differ by subpopulation and by the measures that define SEP. Current wealth was most strongly associated with BMI among white non-Hispanic women in another large U.S. cohort [22]. Two recent studies from presumably racially/ethnically homogeneous populations in Finland [23] and Sweden [24] reported relatively weak or null associations between childhood SEP and adult weight status, further emphasizing the fact that socioeconomic and racial/ethnic disparities are not universal, but are socially contingent [25]. Notably, these studies and ours arrived at similar findings despite differences in how childhood SEP was measured. The Scandinavian studies used multiple markers of childhood SEP, including parental education [23], childhood economic “difficulties” [23], and household breadwinner’s occupation [24], all recalled by respondents. Comparison between studies that use identical measures and target similar populations is an area for future investigation for more refined understanding of the role of socioeconomic factors in influencing health over the life-course.

Associations between excessive gestational weight gain and adult obesity have been documented by others [3,26,27]. Our findings add to the considerable evidence that prevention of excessive GWG should be an important maternal health objective. In addition to long-term maternal weight, excessive GWG has been associated with adverse pregnancy, birth, and child outcomes [2,4,5].

We identified no existing literature on the relationship between childhood SEP and GWG. Two studies connected adult SEP and GWG, which provided contradictory findings. Greater education was associated with more excessive GWG in an American study [28] and with less excessive GWG among Swedish women, but only those with normal prepregnancy BMI [29]. Elsewhere, neighborhood socioeconomic disadvantage was associated with inadequate, but not excessive, GWG [30]. Here, we found inconsistent and relatively weak associations between childhood SEP and excessive GWG.

None of our childhood socioeconomic disadvantage variables were significantly associated with both excessive GWG and adult obesity, and we found little evidence that excessive GWG is a mediator of any effects of childhood SEP on midlife obesity among parous American women. However, this does not rule out the possibility of undetected partial mediation. Importantly, our measures of childhood SEP were collected at baseline (age 14–21 in the NLSY 1979). Direct measures of socio-economic disadvantage in early childhood might have yielded different findings.

While the NLSY 1979 cohort represents a racially/ethnically diverse population, statistical power to detect a total effect of some childhood SEP variables was limited among black and Hispanic women, because, for many SEP measures, a striking majority experienced childhood socioeconomic disadvantage. So few parents of black or Hispanic women had attainted formal higher education that exposure stratification into multiple ordered categories was impractical, despite being a preferred approach [8]. This illustrates how the cumulative disadvantage experienced by racial and ethnic minorities in the United States [31] has implications not only for social policy but also for social epidemiology research [32]. While this cohort is contemporary regarding midlife obesity status, employment and education of the previous generation might not ideally indicate socioeconomic position, particularly among racial/ethnic minorities, for whom mid-20th century segregation and racism might have limited socioeconomic opportunities that education might have otherwise afforded [33]. We balanced this limitation by using multiple measures to define socioeconomic disadvantage and using different cutpoints to create binary variables. Data on other measures, such as wealth [34], were unavailable and might have been relevant.

Maternal weight data were self-reported, and GWG information was recalled for births prior to 1986. Despite these limitations, the NLSY 1979 cohort offers extensive, prospectively collected socioeconomic data, allowing for comparisons of multiple SEP measures. Also, this analysis took into account excessive GWG events over a woman’s lifetime, rather than a single birth, as usually considered [3,26,27].

While MSMs have been used to make causal inference in a variety of observational settings, it is plausible that not all confounding variables were measured and that treatment and censoring models were incorrectly specified. For example, energy balance - a likely shared determinant of excessive GWG and obesity - was not recorded in the NLSY 1979 cohort. Thus, our findings represent potentially interesting associations that should be interpreted within the existing body of evidence, rather than precise estimates of causal effects.

Others have also found that the relationship between life course SEP and health is sensitive to the measure of socioeconomic position used [8,34,35], and that race/ethnicity modifies adult SEP-health associations [36]. We add to these findings and encourage future researchers to characterize different dimensions of SEP over the life-course. Nuanced approaches to characterizing exposure may be needed for effective health promotion against multi-factorial, complex conditions, such as obesity.

## Conclusions

In summary, we found disparities by race/ethnicity in both childhood SEP and adult obesity and that race/ethnicity was an effect measure modifier of the associations between childhood SEP, excessive GWG, and adult obesity. Low parental educational attainment was associated with adult obesity among non-black, non-Hispanic women and non-black Hispanic women, but this relationship did not hold for black non-Hispanic women. Childhood SEP was not consistently associated with experiencing excessive GWG in any racial/ethnic group. In agreement with prior reports, experiencing excessive GWG in at least one birth was associated with obesity in adulthood. However, there was no evidence that excessive GWG mediates the relationship between childhood SEP and adult obesity. Therefore, while excessive weight gain in pregnancy remains a pressing public health problem in need of effective prevention, we did not identify evidence that pregnancy-related weight gain contributes to even greater levels of adulthood obesity among women who experienced disadvantage in childhood.

## Abbreviations

BMI: Body mass index
CI: Confidence interval
GWG: Gestational weight gain
MSM: Marginal structural model
NLSY: National Longitudinal Survey of Youth
RR: Risk ratio (cumulative incidence ratio)
SEP: Socioeconomic position
